# Removal of Cypermethrin from Water by Using *Fucus Spiralis* Marine Alga

**DOI:** 10.3390/ijerph16193663

**Published:** 2019-09-29

**Authors:** Violeta Năstuneac, Mirela Panainte-Lehăduș, Emilian Florin Moșneguțu, Simona Gavrilaș, Gabriela Cioca, Florentina-Daniela Munteanu

**Affiliations:** 1 “Vasile Alecsandri” University of Bacău, Faculty of Engineering, 157 Mărăşeşti Str., 600115 Bacău, Romania; violeta10nvn@gmail.com (V.N.); mirelap@ub.ro (M.P.-L.); emos@ub.ro (E.F.M.); 2 “Aurel Vlaicu” University of Arad, Faculty of Food Engineering, Tourism and Environmental Protection, 2-4 E. Drăgoi Str., 310330 Arad, Romania; simona2213@yahoo.com; 3 Preclinical Department, Faculty of Medicine, Lucian Blaga University of Sibiu, 550024 Sibiu, Romania; gabriela.cioca@ulbsibiu.ro

**Keywords:** alpha-cypermethrin, brown marine algae, detoxification, isotherms, kinetics

## Abstract

Alpha-cypermethrin is a synthetic pyrethroid that was extensively used for insect control, since the early 1980s. However, it is known that its presence in the environment has toxic effects on humans and aquatic life forms. For this reason, it is commendable for it to be removed completely from the contaminated environment. In this study, we evaluated the adsorption capacity of a marine alga for the removal of cypermethrin from water. The adsorption experiments were performed based on the batch equilibrium technique. The samples containing the pesticide were analyzed using gas chromatography with an electron capture detector, after liquid-liquid extraction in hexane. The results obtained from the kinetic adsorption studies showed that the equilibrium time was attained after 40 min. The adsorption parameters at equilibrium concentrations, obtained through the Langmuir, Freundlich, and Temkin models, showed that the used brown marine alga has a maximum amount of adsorbed cypermethrin of 588.24 µg/g. The correlation coefficients obtained for each model prove that the Langmuir model best fits the experimental data.

## 1. Introduction

Nowadays, the most used pesticides are synthetic pyrethroids, as they are not only more cost-effective but also less toxic to the vertebrate wildlife [1] compared to other pesticides (organochlorines, carbamates, and organophosphates). Synthetic pyrethroids were developed after the 1970s to overcome the photo-liability of natural pyrethrins, which were extracted from the flower-heads of *Chrysanthemum cinerariaefolium*.

The pyrethroids were physiochemically characterized in an extensive review [2], where it was shown that these compounds are highly non-polar molecules, which means a low water solubility, and therefore, they present a high bioaccumulation potential. Another characteristic refers to their hydrolytic stability at acidic and neutral pHs, while in alkaline conditions, some pyrethroids suffer rapid degradation because of the cleavage of the ester bridge of the molecule [2]. The lipophilicity of these compounds was the subject of different-standard laboratory toxicity research to prove that once the pyrethroids enter the aquatic environment, the exposure to the water phase is diminished by the adsorption and degradation processes. However, for a better understanding of their impact on the environment, several studies were performed in the field. The laboratory and the in-field research led to an impressive database with results on the potential effects of the synthetic pyrethroids [3,4,5]. Nowadays, these pesticides are extensively used in hospitals, agriculture, forestry, horticulture, households, and in the textile industry.

Cypermethrin (CYP), (RS)-α-cyano-3-phenoxybenzyl (1RS,3RS;1RS,3SR)-3-(2,2-dichlorovinyl)-2,2-dimethylcyclopropanecarboxylate, is a widely used pyrethroid, as it is known to have a high pesticide activity [6]. It is also used for the preservation of wool and cotton in the textile industry. As a consequence, cypermethrin is frequently detected in surface waters in concentrations ranging from 0.1 to 194 μg/L [7].

However, the presence of the cypermethrin in the environment is not desirable, as was proven by recent studies [6,8,9,10,11,12,13,14]. Moreover, its presence in water represents a concern around the world, and new methods for its removal from water have been proposed [9,15,16,17,18,19]. Among the reported methods for the removal of pesticides, adsorption was found to be the most effective [20].

Different materials can be used as adsorbents, but in the last period, natural materials are becoming more attractive, as these are renewable and low-cost [9,18,19,21,22]. Moreover, their increasing attractiveness is also related to the demand to reduce waste, to capitalize the sub-products from different industries, and to maximize the efficiency of the natural products [23]. 

The present paper focuses on the possibility to use a brown marine alga, *Fucus spiralis*, for the removal of cypermethrin from water. The adsorption of the cypermethrin from the water on *Fucus sp*. was kinetically characterized through focus on the pseudo-first-order and pseudo-second-order models. Equilibrium studies were also carried out, and the data were fitted into Langmuir, Freundlich, and Temkin isotherms.

## 2. Materials and Methods

The brown alga, *Fucus spiralis*, was used as a dried powder with a particle size ranging from 150 to 300 µm. Hexane and cypermethrin were of analytical grade.

An individual stock solution of 200 mg/L cypermethrin was prepared in hexane. For the preparation of the aqueous solutions, a known volume of the cypermethrin solution prepared in hexane was added to a flask, and the hexane was removed by exposure to nitrogen (purity 6.0). Thereafter, the aqueous solutions of the desired concentration were obtained by the addition of distilled water.

The biosorption experiments were performed in the batch by adding 400 mg of alga to the Erlenmeyer flasks containing the cypermethrin aqueous solution. The experiments were performed at neutral pH. A series of Erlenmeyer flasks containing 300 mL of a 200 μg/L α-cypermethrin solution and 400 mg of alga was stirred (400 rpm) on a magnetic multi-stirrer (Velp Scientifica, Italy) at 25 °C. Samples were withdrawn from the stirrer at certain moments and were filtered. The supernatant contained the remaining cypermethrin in water after adsorption on the alga. The remaining cypermethrin was extracted in hexane through the liquid-liquid extraction method.

The detection of cypermethrin was done by using an electron capture detector gas chromatograph (GC-ECD), Shimadzu GC 2010 (Shimadzu Corporation, Kyoto, Japan) equipped with a Zebron column (length—30 m, inner diameter—0.25 mm, film thickness—0.25 µm) (Phenomenex, USA) and an HTA autosampler (HTA, Italy). Before injection into the GC-ECD, all the samples of cypermethrin (prepared in hexane) were centrifuged for 10 min at 9000 rpm (Sigma 2-16 Centrifuge, United Kingdom). The mobile phase consisted of helium (6.0 purity) and nitrogen as make-up gas. The flow rate of the mobile phase was 30 mL/min. The gradient temperature program started from an initial temperature of 60 °C, where it was held for 1 min, then the temperature was raised to 180 °C with 26 °C/min, where it was held for 11 s and afterwards raised to 230 °C with 5 °C/min, where it was held for 18 s, and finally the temperature was raised with 7 °C/min to the final temperature of 270 °C, where it was maintained for 11 min. The detector temperature was 310 °C. The obtained chromatogram presented the specific cypermethrin peaks with retention times at 23.6 ± 0.2 min.

For the external calibration curve (R^2^ = 0.9977), the cypermethrin solutions were prepared in hexane by serial dilution from a 200 mg/L stock solution, obtaining working solutions with concentrations in the range of 50 µg/L to 1000 µg/L (50, 150, 250, 350, 450, 550, 650, 750, 850, 1000 µg/L). The calibration curve was obtained with controls extracted in the same conditions as the samples. 

For the adsorption equilibrium experiments, 40 mg of the powdered *Fucus sp.* were shaken for 120 min with 25 mL aqueous solutions of cypermethrin of 13 different concentrations in the range of 100 to 3500 µg/L (100, 150, 250, 450, 500, 650, 750, 1000, 1500, 2000, 2500, 3000, 3500 µg/L). The mixture was filtered and the residual concentration of the cypermethrin in the filtrate was extracted in hexane and analyzed by GC-ECD. The amount of cypermethrin adsorbed at time *t*, *q_t_*, was calculated as follows:

(1) $$q_{t}=\frac{(C_{0}-C_{t)}*V}{m}$$

where *C*_0_ and *C_t_* (µg/L) are the concentrations of cypermethrin in solution at initial and at time *t*, *V* is the volume of solution (25 mL), and m is the mass of adsorbent (0.04 g). The obtained data were fitted using the following isotherms: Langmuir [24], Freundlich [25], and Temkin [26].

The kinetic studies were performed based on the batch technique. For this purpose, 300 mL of an aqueous solution of 200 µg/L of cypermethrin were stirred with 400 mg of alga. Samples were withdrawn from the stirrer at certain moments, and the remaining cypermethrin in the supernatant was extracted in hexane and used for the GC-ECD analysis. The adsorption capacity of the alga was calculated for each considered contact time.

The obtained experimental data were tested with the pseudo-first-order kinetic model [27] (Equation (2)) and the pseudo-second-order kinetic model [28] (Equation (3)).

(2) $$q_{t}=q_{e}\left(1-e^{-k_{1}t}\right)$$

(3) $$q_{t}=\frac{q_{e}\left(1-e^{-k_{1}t}\right)}{1+q_{e}k_{2}t}$$

where *t* is the contact time (in minutes) between the cypermethrin solution and the alga, *q_t_* (µg/mg) is the amount of cypermethrin adsorbed by a mass unit of adsorbent at time *t*, *q_e_* (µg/mg) is the amount of cypermethrin adsorbed when the equilibrium is attained and *k*_1_ (min^−1^), and *k*_2_ (mg·µg^−1^min^−1^) are the pseudo-first and, respectively, pseudo-second-order rate constants.

## 3. Results and Discussion

### 3.1. Kinetic Modelling

The analysis of cypermethrin in GC-ECD was performed under the described temperature gradient, and the elution time was 23.6 ± 0.2 min. The external calibration curve for cypermethrin was obtained for ten different concentrations (50 µg/L to 1000 µg/L) and is described by a linear dependency.

For the evaluation of the capability of the alga to adsorb the cypermethrin from water, a kinetic study was conducted. From this study, we could evaluate the time needed to reach an adsorption equilibrium. In the present paper, we considered the pseudo-first-order (Lagergren model) [27] and pseudo-second-order [28] kinetic reaction models. 

**Figure 1:**
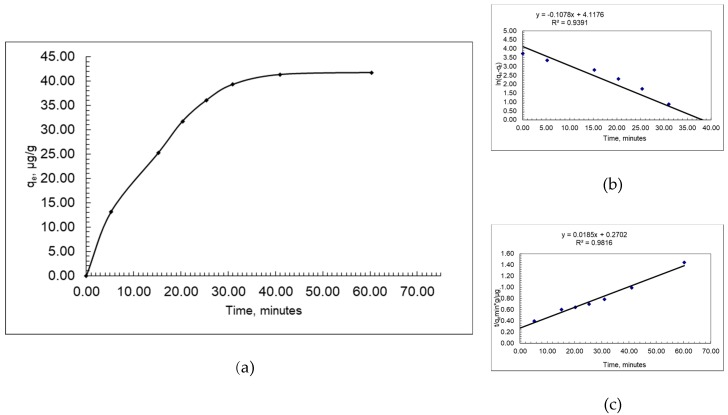
Adsorption (**a**), pseudo-first (**b**), and pseudo-second-order (**c**) kinetics of cypermethrin on *Fucus spiralis.*

Figure 1 presents the adsorption kinetics of the cypermethrin on the *Fucus sp.* Alga, and it can be observed that the equilibrium is reached after 40 min, with no significant changes in the adsorbed amount of cypermethrin after reaching the equilibrium. 

The pseudo-first-order model considers that the adsorption rate is proportional to the number of unoccupied sites, as presented by Equation (2). 

The pseudo-second-order-model can be used to predict if the adsorption is the rate-controlling step (Equation (3)).

The values of the pseudo-first-order rate constant (*k*_1_) and the amount adsorbed at equilibrium (*q_e_*) were calculated (Table S1).

The values obtained for the maximum uptake capacity, 61.41 µg/mg for the pseudo-first-order model, and, respectively, 63.36 µg/mg for the pseudo-second-order model, are similar, a fact that is also described by the correlation coefficients (0.94 and 0.98) (Table S1). It can be concluded that the pseudo-first-order and the pseudo-second-order models well describe the kinetics of the cypermethrin adsorption on the dried and powdered *Fucus spiralis*. 

### 3.2. Isotherm of Biosorption

The isotherms of biosorption are useful for the evaluation of the surface properties and affinity of the alga for cypermethrin and might be used to understand the relationship between the mass of cypermethrin per biosorbent mass and its concentration in the solution. In our studies, three biosorption isotherms for the fitting of the experimental data obtained for the adsorption of cypermethrin on *Fucus spiralis* were considered.

The first considered adsorption isotherm was the one proposed by Langmuir [24]. This model considers the formation of a monolayer of the adsorbate on the surface of the adsorbent. Therefore, this model assumes that the adsorption is stopped after the formation of the monolayer. Considering these assumptions, the Langmuir isotherm is represented by the following equation:

(4) $$q_{e}=\frac{Q_{o}*K_{L}*C_{e}}{1+K_{L}*C_{e}}.$$

After linearization of Equation (4), the following equation is obtained:

(5) $$\frac{1}{q_{e}}=\frac{1}{Q_{o}}+\frac{1}{Q_{o}*K_{L}*C_{e}}$$

where *q_e_* is the amount of adsorbed cypermethrin on the dried powdered alga (µg/g), *Q*_0_ is the maximum monolayer capacity (µg/g), *C_e_* is the equilibrium concentration of cypermethrin (µg/L), and *K_L_* is the Langmuir constant (L/µg).

Another characteristic of the Langmuir isotherm is the fact that from Equation (5) we can define *R_L_* as an equilibrium parameter that is dimensionless and is described by Equation (6):

(6) $$R_{L}=\frac{1}{1+\left(1+K_{L}*C_{0}\right)}$$

where *C*_0_ is the equilibrium concentration of cypermethrin (µg/L) and *K_L_* is the Langmuir constant (L/µg).

In the case that *R_L_* > 1, then the adsorption is not possible, if *R_L_* = 1, then the adsorption is linear, if 0 < *R_L_* < 1, the adsorption is favorable, while for *R_L_* = 0 the adsorption process is irreversible [29]. 

**Figure 2:**
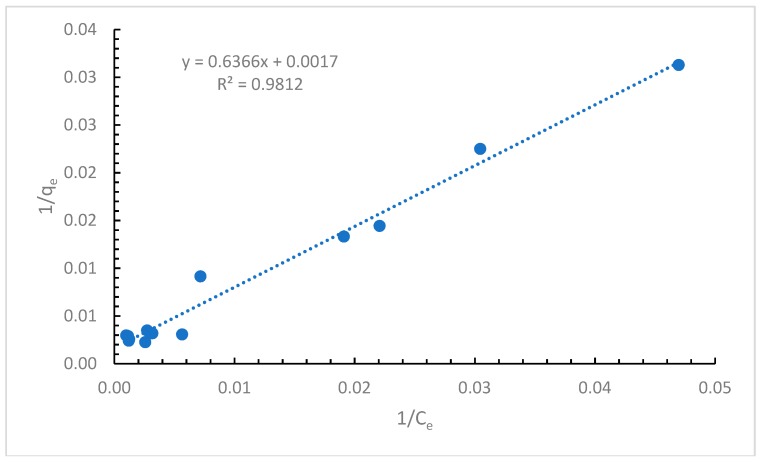
Langmuir adsorption isotherm.

The experimental results fitted with the Langmuir isotherm are presented in Figure 2.

The straight line obtained with a good correlation coefficient proves that this model is reliable. 

Based on the data calculated (Table S2), the value of 0.79 obtained for *R_L_* is between 0 and 1, which indicates that the adsorption of cypermethrin onto the alga is favorable and the maximum monolayer coverage is 588.24 µg/g. 

As a second model was considered the Freundlich adsorption isotherm described by the equation:

(7) $$q_{e}=K_{F}C_{e}^{\frac{1}{n}}$$

with its linearized form presented by Equation (8):

(8) $$logq_{e}=logK_{F}+\frac{1}{n}logC_{e}$$

where *q_e_* is the amount of adsorbed cypermethrin on the dried powdered alga (µg/g), *C_e_* is the equilibrium concentration of cypermethrin (µg/L), *n* is the adsorption intensity, and *K_F_* is the Freundlich constant (µg/L).

**Figure 3:**
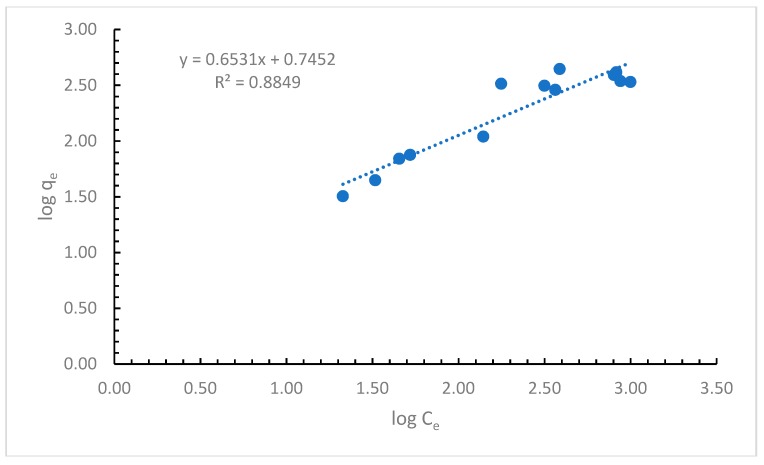
Freundlich adsorption isotherm.

The obtained experimental results were fitted with the linearized equation of the Freundlich adsorption isotherm. The obtained curve is presented in Figure 3. 

Considering this model, in the case that *n* has a value between 1 and 10, then the adsorption of cypermethrin on the alga is favorable. 

The calculated parameters for the Freundlich isotherm (Table S3) show that *n* has a value of 1.53, which indicates a favorable adsorption of the cypermethrin on the used alga. The value obtained for the Freundlich constant, 5.56 µg/L, refers to the adsorption capacity. 

The third model used in this study is based on the Temkin adsorption isotherm that considers the adsorbent–adsorbate interactions [26]. The equation that describes this model is below:

(9) $$q_{e}=\frac{RT}{b_{T}}ln\left(A_{t}*C_{e}\right)$$

(10) $$q_{e}=\frac{RT}{b_{T}}lnA_{t}+\frac{RT}{b_{T}}lnC_{e}$$

and if it is considered that

(11) $$B=\frac{RT}{b_{T}}$$

then,

(12) $$q_{e}=B*lnA_{t}+B*lnC_{e}$$

where *A_T_* is the Temkin isotherm equilibrium binding constant (L/µg), *b_T_* is the Temkin isotherm constant, *R* is the universal gas constant (J/mol/K), *T* is the temperature in *K*, and *B* is the constant related to heat of the sorption process (J/mol).

**Figure 4:**
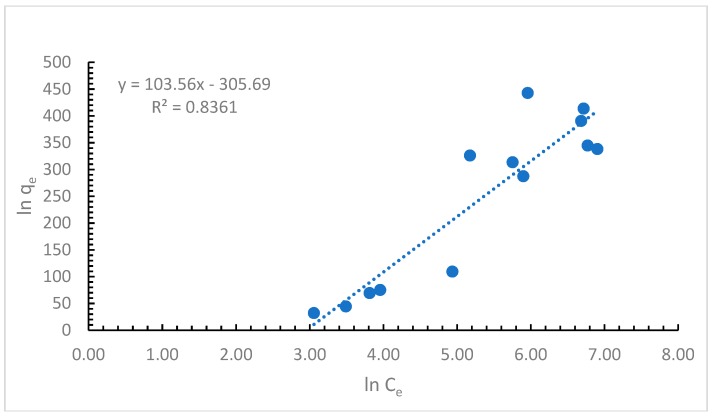
Temkin adsorption isotherm.

From the Temkin isotherm plot presented in Figure 4, the values from Table 1 were calculated.

The values presented in Table 1 are an indication of physical adsorption. The value obtained for B, 103.56 J/mol indicates a strong interaction between the cypermethrin and the alga. The value of R^2^ for the experimental data fitted with the Temkin isotherm shows that this model is not as good as the other two isotherm models.

After examining the results obtained by applying the Langmuir, Freundlich, and Temkin isotherms, it can be concluded that the most appropriate to be used is the Langmuir isotherm, as it shows the highest correlation coefficient of all three. As the Langmuir model is considered the most appropriate model to be used in our studies, the maximum biosorption capacity of other adsorbents used for the retention of cypermethrin are presented in Table 2.

The literature that reports values for the biosorption of cypermethrin from water by different adsorbents shows that the maximum biosorption capacity of *Fucus spiralis* is one of the highest values (Table 2), which makes the chosen alga a promising candidate for the removal of cypermethrin from water. Further studies will be performed to prove its adequacy for polluted water remediation.

## 4. Conclusions

In this study, the kinetic and equilibrium data for the batch biosorption process of cypermethrin were obtained using dried and powdered *Fucus spiralis*. 

The necessary time to reach the adsorption equilibrium was 40 min, with no significant changes in the amount of adsorbed cypermethrin after reaching the equilibrium. Moreover, the results show that both considered kinetic models, pseudo-first-order (correlation coefficient, R^2^, 0.94) and pseudo-second-order (correlation coefficient, R^2^, 0.98), well describe the kinetics of the cypermethrin adsorption process on the alga. The maximum uptake capacities calculated by applying the models show values that are in good agreement (61.41 µg/mg for the pseudo-first-order model, respectively, 63.36 µg/mg for the pseudo-second-order model).

The experimental data were also fitted using three different biosorption models, and it was found that the Langmuir model best fitted the biosorption data. The maximum coverage calculated from this model is 588.24 µg/g. Although the use of the dried and powdered *Fucus spiralis* for the removal of cypermethrin from water needs further studies, this alga shows potential for the treatment of pyrethroid polluted water.

## Figures and Tables

**Table 1:** Temkin isotherm constants obtained for the cypermethrin adsorption.

A_T_ (L/µg)	b_T_	B (J/mol)	R^2^
75.85	23.92	103.56	0.84

**Table 2:** Maximum biosorption capacity based on the Langmuir isotherm by different adsorbents for cypermethrin.

Adsorbent	Q_0_ (µg/g)	Reference
*Fucus spiralis*	588.24	Present study
Cork (1–2 mm)	303.00	[30]
Activated carbon	186.00	[30]
Cork (3–4 mm)	136.00	[30]
Carbon aerogel	66.22	[31]
Carbon xerogel	61.73	[31]

## Supplementary Materials

The following are available online at https://www.mdpi.com/1660-4601/16/19/3663/s1, Table S1: Pseudo-first-order and pseudo-second-order model parameters, Table S2: Langmuir isotherm constants obtained for the cypermethrin adsorption, Table S3: Freundlich isotherm constants obtained for the cypermethrin adsorption.
