# Supplementary material for: Removal of Cypermethrin from Water by Using Fucus Spiralis Marine Alga
Source: Int J Environ Res Public Health. 2019 Sep 29;16(19):3663. doi: 10.3390/ijerph16193663 (PMC6801688; doi:10.3390/ijerph16193663)
Supplement: Supplementary file 1 [file ijerph-16-03663-s001.pdf]

Table S1. Pseudo-first-order and pseudo-second-order model parameters.

Pseudo-first-order model		
**k_1 (min^{-1})**	**q_e (μg/mg)**	**R^2**
6.43	61.41	0.94
**Pseudo-second-order model**		
**k_2 (μg $mg^{-1}min^{-1}$)**	**q_e (μg/mg)**	**R^2**
0.013	63.36	0.98

Table S2. Langmuir isotherm constants obtained for the cypermethrin adsorption.

Q_0 (μg/g)	K_L (L/μg)	R_L	R^2
588.24	0.0027	0.79	0.98

Table S3. Freundlich isotherm constants obtained for the cypermethrin adsorption.

n	K_F (μg/L)	R^2
1.53	5.56	0.88
